# Bone-Remodeling Transcript Levels Are Independent of Perching in End-of-Lay White Leghorn Chickens

**DOI:** 10.3390/ijms16022663

**Published:** 2015-01-23

**Authors:** Maurice D. Dale, Erin M. Mortimer, Santharam Kolli, Erik Achramowicz, Glenn Borchert, Steven A. Juliano, Scott Halkyard, Nick Sietz, Craig Gatto, Patricia Y. Hester, David A. Rubin

**Affiliations:** 1 School of Biological Sciences, Illinois State University, Normal, IL 61701, USA; E-Mails: mddale@ilstu.edu (M.D.D.); mortimr2@illinois.edu (E.M.M.); srkolli@gmail.com (S.K.); eachramowicz@gmail.com (E.A.); sajulian@ilstu.edu (S.A.J.); sdhalkyard@gmail.com (S.H.); nseitz@ilstu.edu (N.S.); cgatto@ilstu.edu (C.G.); 2 Department of Biology, University of South Alabama, Mobile, AL 36688, USA; E-Mail: borchert@southalabama.edu; 3 Department of Animal Sciences, Purdue University, 125 South Russell St, West Lafayette, IN 47907, USA; E-Mail: phester@purdue.edu

**Keywords:** laying hen, qPCR, bone-specific transcripts, bone-remodeling, signaling network

## Abstract

Osteoporosis is a bone disease that commonly results in a 30% incidence of fracture in hens used to produce eggs for human consumption. One of the causes of osteoporosis is the lack of mechanical strain placed on weight-bearing bones. In conventionally-caged hens, there is inadequate space for chickens to exercise and induce mechanical strain on their bones. One approach is to encourage mechanical stress on bones by the addition of perches to conventional cages. Our study focuses on the molecular mechanism of bone remodeling in end-of-lay hens (71 weeks) with access to perches. We examined bone-specific transcripts that are actively involved during development and remodeling. Using real-time quantitative PCR, we examined seven transcripts (COL2A1 (collagen, type II, alpha 1), RANKL (receptor activator of nuclear factor kappa-B ligand), OPG (osteoprotegerin), PTHLH (PTH-like hormone), PTH1R (PTH/PTHLH type-1 receptor), PTH3R (PTH/PTHLH type-3 receptor), and SOX9 (Sry-related high mobility group box)) in phalange, tibia and femur. Our results indicate that the only significant effect was a difference among bones for COL2A1 (femur > phalange). Therefore, we conclude that access to a perch did not alter transcript expression. Furthermore, because hens have been used as a model for human bone metabolism and osteoporosis, the results indicate that bone remodeling due to mechanical loading in chickens may be a product of different pathways than those involved in the mammalian model.

## 1. Introduction

Skeletal bones and teeth are unique systems that rely on calcification of an extracellular matrix. Parathyroid hormone (PTH) regulates the inorganic calcium matrix in bone by stimulating the parathyroid hormone receptor (PTHR) [1]. Upon PTHR stimulation, osteoclasts become activated, resulting in resorption of trabecular and cortical bone [2]. Cortical (alias compact) bone is the structural outer region of bone, which also makes up the shaft of long bones. Trabecular (alias spongy) bone is located in the epiphysis of long bones and the interior of vertebrae and is organized as a loose network. In addition to cortical and trabecular bone, female birds that are reproductively active have an additional reservoir of bone calcium [3]. At the onset of sexual maturity, which occurs at about 18 weeks in white leghorn pullets, estrogen simulates the production of the medullary bone of the hen skeleton [3]. Thin spicules of loosely-woven medullary bone located in the marrow cavity provide an easily accessible and quick source of calcium. Upon PTHR stimulation, calcium is sequestered from medullary bone, from where it is used by the uterus for eggshell formation [3,4].

Activation of bone remodeling occurs due to microfractures and mechanical loading, which is sensed by osteocytes. Although Transforming growth factor β (TGF-β) is necessary for osteoblast formation, during activation of bone remodeling, TGF-β production is inhibited [2]. When PTH binds to the PTHR on osteoblasts and osteocytes, it stimulates the activation of intracellular pathways and the secretion of RANK ligand (RANKL) [5]. The secreted RANKL binds to preosteoclasts that are induced to differentiate into osteoclasts. Osteoclast activation results in the resorption of bone matrix that increases serum calcium and phosphate. Osteoclast activity is inhibited by the secreted ligand osteoprotegerin (OPG), which binds to RANKL and prevents the binding of RANKL to RANK [2,5]. In addition to inactivating the resorption phase of bone remodeling, OPG also activates a reversal phase, which is characterized by an increase expression of signals necessary for osteoblast recruitment. Finally, during bone remodeling due to mechanical loading, PTH inhibits sclerostin production by osteocytes. Therefore, PTH blocks sclerostin from inhibiting bone formation, while calcitonin (antagonist of PTH) increases sclerostin production, thus completing a negative feedback system [2].

Significant bone diseases occur because of unbalanced bone remodeling. For instance, osteoporosis is a common and prevalent disorder resulting in the loss of bone mass and strength, increasing the probability for bone fractures [6]. Many factors contribute to the formation of osteoporotic bones, including impaired bone formation during development, estrogen deficiency and lack of exercise [6]. Lack of exercise contributes to increased bone fractures in all vertebrates, including caged laying hens. The pathology of osteoporosis becomes severe in cage-laying hens at the end of lay (71 to 80 weeks of age), with roughly 30% of the hens having one or more broken bones [3].

Laying hens, responsible for the production of table eggs, experience a progressive loss of bone strength and mass as they age, due to the calcium requirements for eggshell formation. During the switch to medullary bone production, structural bone continues to undergo resorption, while the medullary bone is used as a calcium reservoir for eggshell formation. In conventionally-caged hens, the process of structural bone loss is accelerated due to the constraints of the housing unit used in many commercial operations, where the opportunity for exercise is limited [3]. Non-cage housing provides more opportunity for activity, such as jumping on and off perches, thereby improving bone strength [3,7].

Osteoporosis in white leghorns results in economic loss to the egg industry worldwide and is the cause of 20% to 35% of all mortalities during the egg laying cycle of caged hens [8,9,10]. Furthermore, hens with fractured osteoporotic bones experience pain [11]. Providing perches inside cages may increase load on weight-bearing bones to induce osteoblast-mediated bone formation. Thus, providing perches mounted above the caged floor may give hens the opportunity to gain more exercise and potentially put stress on bones, allowing for increased bone remodeling. Although little is known of the mechanisms involved in exercise-induced skeletal improvement in laying hens, the effects of perches have been shown to minimize bone loss in load-bearing leg bones [3,12,13,14,15,16,17,18,19,20,21].

**Figure 1:**
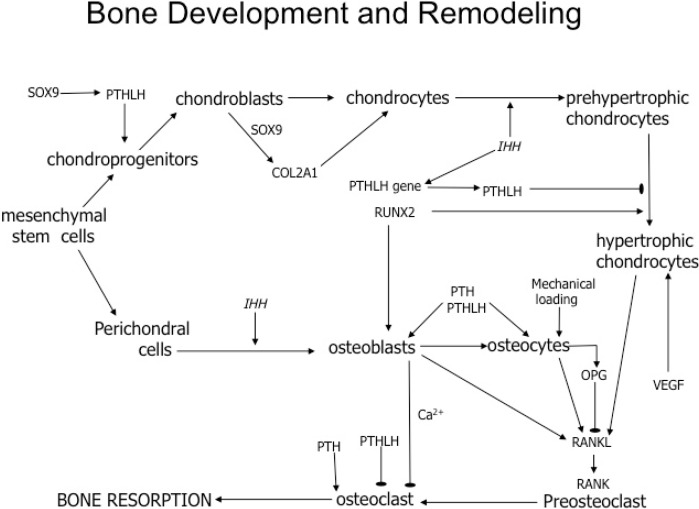
Summary of cellular events and transcription factors that regulate bone development and remodeling. Mesenchymal stem cells give rise to pathways for chondrogenesis and osteogenesis. During chondrocyte development and maturation, SOX9 promotes the expression of PTHLH [22] and COL2A1 (collagen, type II, alpha 1) [23]. When Indian hedgehog (IHH) is secreted by chondrocytes, it not only induces further development of prehypertrophic chondrocytes, but also increases the expression of PTHLH [24]. Subsequently, PTHLH regulates the differentiation of prehypertrophic chondrocytes into hypertrophic chondrocytes, while runt-related transcription factor 2 (RUNX2) acts on prehypertrophic chondrocytes, stimulating their development into hypertrophic chondrocytes [24,25]. During endochondral bone development, hypertrophic chondrocytes are stimulated by vascular endothelial growth factor (VEGF), which promotes vascularization of the cartilage [25]. RANKL is secreted by hypertrophic chondrocytes to recruit osteoclasts to the region of cartilage development. The recruited osteoclast develop the marrow region of the bone. Once osteoblasts arrive at the vascularized cartilage, they begin depositing minerals [25] and subsequently mature into osteocytes, which become imbedded in the lacunae of trabecular and cortical bone. To regulate bone remodeling in vertebrates, RANKL expression by osteoblasts and osteocytes is necessary for osteoclast progenitors to differentiate into osteoclast by binding to the RANK receptor [26]. Osteocytes also regulate osteoclast activity by expressing OPG, which acts as a decoy that binds directly to RANKL [26]. Thus, OPG inhibits RANKL from binding to the receptor on osteoclasts and preosteoclasts, resulting in the inhibition of their activity and differentiation. Osteoclast regulation occurs directly and indirectly by PTH, which binds and activates the PTH/PTHLH receptor. Although the PTH/PTHLH type-1 receptor (PTH1R) is common throughout all vertebrates, birds and fish (and not humans) express a PTH/PTHLH type-3 receptor (PTH3R). While PTH1R is primarily activated by PTH for calcium regulation throughout all vertebrates, it appears that the PTH3R in fish is primarily activated in PTHLH, whereas the activation of PTH3R in birds is not fully understood [27]. In vertebrates, bone resorption occurs when PTH directly activates the PTH/PTHLH receptors on osteoclasts [28]. However, when PTH binds to PTH/PTHLH receptors on osteoblast, calcium is secreted, and the calcium released is sensed by calcium-sensing receptors (CaSR) on the surface of osteoclast [28]. Osteoclast activity is inhibited by the calcium secreted by osteoblasts and by PTHLH, but the mechanism is not completely understood [28]. Osteoclasts in medullary bone are resistant to many factors that inhibit their activity in trabecular and cortical bone [4]. (Abbreviations: SOX9, SRY (sex-determining region Y)-box 9; PTHLH, parathyroid hormone like-hormone; COL2A1, collagen, type II, alpha 1; IHH, Indian Hedgehog, NM_204957; RUNX2, Runt-related transcription factor 2, NM_204128; VEGF, vascular endothelial growth factor, AB011078; RANKL, receptor activator of nuclear factor kappa-B ligand; OPG, osteoprotegerin; PTH, parathyroid hormone; PTHLH, PTH-like hormone; PTH1R, PTH/PTHLH type-1 receptor; PTH3R, PTH/PTHLH type-3 receptor; CaSR, calcium-sensing receptor, XM_416491.4).

In this study, we determined mRNA expression in 71-week-old white leghorn hens with or without access to perches at different stages of their life cycle. We examined differences in transcript expression between the parathyroid hormone type-1 receptor (PTH1R) *vs*. the parathyroid hormone type-3 receptor (PTH3R), as well as the RANKL/OPG ratio, which can provide insight into the activity of osteocytes and osteoclasts in response to mechanical loading on the bone. We also examined transcript expression for Sry-related high mobility group box (SOX9) and PTH-like hormone (PTHLH, alias PTHrP), which are necessary for endochondrogenesis. We used two weight-bearing bones (tibia and femur) and a non-weight-bearing bone (III carpometacarpal, alias phalange). The findings will be used to compare bone-remodeling pathways in chickens and humans (Figure 1).

In our first hypothesis, we anticipate that the RANKL/OPG ratio will be decreased in chickens that have access to perches during their entire life cycle, as compared to hens that never have access to perches. The RANKL/OPG ratio expression provides detail into the overall level of osteoclast activity, where a higher ratio might occur during the resorption phase of bone remodeling and is associated with osteoclastogenesis and osteoclast activation; that is, OPG secretion by osteocytes decreases the RANKL/OPG ratio to inhibit the recruitment and activity of osteoclasts. SOX9 is downregulated in the hypertrophic cartilage and further during ossification of endochondral ossification. Because the bones of these 71-week-old hens are already ossified, our second hypothesis is that perch access will not affect the expression of SOX9.

The third hypothesis we propose is that PTHLH will be involved in calcium regulation through either PTH1R or PTH3R. Although PTHLH activates both receptors, in zebrafish and chicken, it has been shown to preferentially activate the PTH3R in zebrafish [27,29]. We hypothesize that PTHLH expression will not be affected by perch access, because PTHLH performs several distinctive functions within bone. In addition, we hypothesize that PTH1R activation will be the primary receptor expressed over PTH3R.

Finally, we propose that bone turnover will be higher in weight-bearing bones, as compared to the non-weight-bearing phalange. Because cage-laying hens rely heavily on the musculoskeletal component of the leg to jump on and off the perch, we anticipate the greatest difference in transcript expression in the femur and tibia, as compared to the phalange. Because the phalange is not directly involved in the effects due to mechanical load (a perch) and medullary bone and diet supply the overarching source of calcium, we hypothesize that the phalange is likely to show no significant treatment effect. Overall, our findings indicate that the addition of a perch did not enhance the molecular mechanisms used for bone formation as a result of mechanical loading. This may be due to the already accelerated bone turnover in egg-laying hens. Thus, our data do not support the model, as we expected.

## 2. Results

All primers and amplicons were validated by subcloning and DNA sequencing (Table 1). Furthermore, each sequenced amplicon was analyzed for the corresponding amino acid sequence using http://web.expasy.org/translate/. GAPDH yielded an amplicon 210 bases with a 69 amino acid sequence match (100% identity). COL2A1 yielded an amplicon of 200 bases with a 66 amino acid sequence match (97% identity). SOX9 yielded an amplicon of 293 bases with a 97 amino acid sequence match (99% identity). PTHLH yielded an amplicon of 151 bases with a 50 amino acid sequence match (99% identity). PTH3R yielded an amplicon of 94 bases with a 29 amino acid sequence match (93% identity). OPG yielded an amplicon of 199 bases with a 46 amino acid sequence match (91% identity). RANKL yielded an amplicon of 298 bases with a 99 amino acid sequence match (99% identity). Table 1 indicates the amplicon size and accession number for each transcript analyzed.

The first set of analyses were performed to determine if the RANKL/OPG ratio would decrease in hens provided access to perches throughout their life cycle as compared to no perch access or access to perches only during the pullet or laying phases. The ratio results indicated no effect due to treatment, bone or the interaction of treatment with bone (Table 2). Individually, neither RANKL nor OPG expression were different among perching treatments nor bone types (Table 2).

Our hypothesis that SOX9 expression would be limited in ossified bone and not affected by perch access was supported by a lack of a treatment effect (Table 2). Likewise neither bone nor its interaction with treatment were significant (Table 2).

**Table 1:** Primers used during QRT-PCR. NT, nucleotide.

Transcript	Primer Pair (5' to 3')	Amplified NT Location (Amplicon Size)	Accession Number
GAPDH	F: GCACCACCAACTGCCTGGCACCCTTG	425–634 (210)	ENSGALT00000023323
	R: GGATGACTTTCCCCACAGCCTTAGCAGC		
COL2A1	F: CATCCTCATCCAGGGATCCAAC	3533–3733 (200)	AF452711
	R: ACTCCTGATCGGCTCCGCCAATGT		
SOX9	F: AAATGACAGAAGAACAGGACAAATG	293–585 (293)	ENSGALT00000038513
	R: CTTCACGTGGGGTTTGTTCTTG		
PTHLH	F: TCAGAGCACCAGCTACTGCATGAC	136–286 (151)	NM_205338
	R: CCTAAGCCTGCTACCAACACAA		
PTH1R	F: GCACTAGAAACTACATCCACATGC	521–754 (234)	DQ914925.1
	R: AGTAGTAATTGGTTGCCAGGAAGT		
PTH3R	F: ACCAGCTGCCTCCCAGAATGGGAT	111–204 (94)	XM_425837
	R: TGAAGTCGTAGATGTAGTCAGGGC		
OPG	F: AGACTGGAACAGCAACGACGAG	272–470 (199)	ENSGALT00000030811
	R: GACAGACTGCTTTGGATGACGT		
RANKL	F: AGGAGGTGAAGTTAATGCCAGAAT	101–398 (298)	ENSGALT00000027412
	R: AGTTTCCCATCACTGAACGTCATA		

The third hypothesis tested whether there would be a difference in expression of PTH1R compared to PTH3R (*i.e*., was the PTH1R preferentially used for bone turnover). Expression of PTH1R was not affected by treatment, bone or the treatment by bone interaction (Table 2). Although we proposed PTH1R to be the primary PTH receptor expressed during chicken bone turnover in response to calcium secretion, our results indicate that PTH3R responded in the same fashion as PTH1R due to treatment, bone and the treatment-bone interaction (Table 2). There was no difference in the expression of PTHLH due to treatment, bone or the treatment by bone interaction (Table 2).

**Table 2:** Statistical analysis of transcripts data (F-statistic and *p*-value).

Transcript	Treatment	Bone	Interaction
RANKL/OPG RATIO	*F*_3,27_ = 1.81, *p* = 0.17	*F*_2,64_ = 1.48, *p* = 0.25	*F*_6,64_ = 1.09, *p* = 0.39
SOX9	*F*_3,32_ = 0.24, *p* = 0.87	*F*_2,64_ = 1.12, *p* = 0.34	*F*_6,64_ = 0.76, *p* = 0.61
PTH1R	*F*_3,32_ = 0.59, *p* = 0.63	*F*_2,64_ = 2.47, *p* = 0.10	*F*_6,64_ = 0.92, *p* = 0.50
PTH3R	*F*_3,32_ = 0.49, *p* = 0.70	*F*_2,64_ = 0.49, *p* = 0.61	*F*_6,64_ = 1.13, *p* = 0.37
PTHLH	*F*_3,32_ = 0.22, *p* = 0.88	*F*_2,64_ = 0.07, *p* = 0.94	*F*_6,64_ = 1.25, *p* = 0.30
COL2A1	*F*_3,32_ = 1.73, *p* = 0.18	*F*_2,64_ = 4.25, *p* = 0.02	*F*_6,64_ = 0.96, *p* = 0.46

The final hypothesis tested whether the non-weight bearing wing bone (the phalange) would respond differently to perch access compared to the leg bones. COL2A1 was utilized as an additional control gene. There was no effect due to treatment or the treatment and bone interaction (Table 2); however, a difference in COL2A1 expression occurred among bones (Table 2). COL2A1 expression was lowest for the phalange, intermediate for the tibia and greatest for the femur (Table 3). The pairwise difference between phalange and femur was significant by the Tukey–Kramer test (*t*_32_ = 2.42, adjusted *p* = 0.05; Table 3). There were no differences for all other transcripts measured in the phalange *vs.* other bones. Least squares means for all transcripts in all bone-treatment combinations are reported in Table A1.

**Table 3:** Expression of the COL2A1 difference among bones (least squares means ± SE (standard error)). Least squares means associated with the same letter are not significantly different at an experiment-wise α = 0.05, by the Tukey–Kramer test.

Phalange	Tibia	Femur
A 0.54 ± 0.06	AB 0.68 ± 0.06	B 0.79 ± 0.10

## 3. Discussion

Osteoporosis in chickens is a serious disease that reduces livability, causing economic losses to the egg industry. To better understand the molecular mechanism of bone turnover, we compared seven factors that regulate weight bearing and non-weight bearing bones. All qPCR factors were subcloned and sequenced to demonstrate that we were analyzing the orthologs of intent. In addition, we designed our study to investigate chickens using the same mechanisms of bone remodeling as the mammalian model (Figure 1).

We proposed that the RANKL/OPG ratio would decrease as a result of an increase in perching activity. In the mammalian model, exercise that induces mechanical strain prevents the recruitment and activation of osteoclasts by secreting OPG. In addition, RANKL secretion increases in humans when mechanical unloading is occurring [30]. However, in the current study, perch access did not affect the RANKL/OPG ratio. This result is not likely due to poor perch usage, as the nighttime and daytime use of perches by hens of the current study averaged 89% and 14.7%, respectively [31], and the hens responded to perch use with increased bone mineral density of the femur, keel, humerus, radius, ulna and phalange, as compared to hens without perches as adults [32]. Furthermore, early access to perches throughout the pullet phase (hatch to 17 weeks of age) did not facilitate the use of perches as adults. Specifically, hens with no access to perches early in life used the adult perch more during the laying phase than those hens with prior perching experience [31]. Future studies should be conducted that encourage or require regular utilization of the perch by all hens.

Another deviation from the mammalian model may be due to the evolution of PTH receptors. Chicken PTH1R and PTH3R are co-orthologs to PTH receptors in teleosts. In mammals and teleosts, PTH and PTHLH activate the PTH1R nearly equivalently for calcium regulation. Because mammals only express a single ortholog of PTH1R, we theorized that the PTH1R would be the preferential receptor utilized for calcium homeostasis/regulation in adult laying hens. Previous studies have investigated the affinity of these receptors in chickens, but no molecular studies have examined these receptors during bone turnover. In our study, we were unable to determine differences in expression between PTH1R and PTH3R.

SOX9 is directly involved in cartilage patterning and hypertrophic chondrocyte development (Figure 1), and the lack of a response to perch access as adults was an anticipated result. Chickens at 71 weeks of age are no longer developing cartilage around the bone. In the mammalian model for bone turnover, SOX9 expression is limited to adult bone maintenance.

PTHLH performs several crucial roles in bone, such as promoting endochondral ossification, where the cartilage template is replaced by bone. Specifically, PTHLH has an anabolic effect by regulating endochondral bone formation by promoting the recruitment and survival of osteoblasts. PTHLH ligand activates the PTH receptors in mammals and inhibits the apoptosis of osteoblast. Similar to PTH, PTHLH extended secretion promotes RANKL secretion of osteoblasts and osteocytes. In our study, no difference in expression of PTHLH was observed as a result of perch access. The functional role of PTHLH during endochondrogenesis in aging hens may be limited.

Medullary bone production may account for the unaltered RANKL/OPG ratio in the aging laying hen as a result of perch access, as this type of bone is unique to avians and crocodiles [3]. Both the femur and the tibia are medullary bones in female avian adults, while the phalange has not been evaluated for whether or not it is a medullary or pneumatic bone. When medullary bone is present, there is high rate of bone turnover [4]; However, osteoclast activity is not specific only to medullary bone, as exposed cortical or structural bone is also resorbed by osteoclasts [3]. Perhaps by the time measurements were made in the current study at 71 weeks of age, the accelerated bone-turnover capabilities may have become exhausted. However, the medullary bone osteoclast may be resistant to inhibitory factors that regulate osteoclast activity in cortical and trabecular bone [4]. In our study, we measured all bone types and cells together. Our future direction will seek to include isolating the specific types of bone and analyzing them individually to determine the difference between types of bone.

There was a significant difference in the expression of COL2A1 in femur when compared to phalange across all treatment groups. COL2A1 is a collagen-specific protein produced in chondroblasts. The reason for the significant expression of COL2A1 in the femur as compared to the phalange of 71-week-old laying hens is unknown. It is reasonable to assume that this upregulation of COL2A1 in the femur as compared to the phalange is unlikely related to cartilage development, as these egg-laying strains of chickens sexually mature at 18 weeks of age, when the growth plate closes. The significant difference in COL2A1 between bones disqualified it as an additional control gene.

We anticipated that phalange would not be significantly accessed as a calcium reservoir, because hens primarily use the calcium in feed and, if inadequate, then the medullary bone as a source of calcium for the formation of the eggshell. Thus, we expected that there would be a difference in transcript expression among the weight-bearing leg bones and the phalange. The phalange did not show any difference compared to the weight-bearing bones, which might be a result of wing flapping during perching. The phalanges, located in the wings, are likely experiencing mechanical strain, as they use their wings for balance when stepping onto and off the perch. In addition, the phalange of hens responded to perch access, as compared to controls with no adult perches, by demonstrating an increase in bone mineral density similar to the femur and the other wing bones [32].

Mechanical loading in bone occurs in response to weight-bearing stress being placed onto the bone. Bone cells’ overall response to mechanical loading is to increase strength in order to manage the additional load. Within bone, mechanical loading alters fluid flow into the extracellular matrix, as well as increases the force on osteocytes. The sensed mechanical force is translated into a biochemical signal, which alters the cell membrane of osteocytes. The osteocyte’s response to mechanical loading is to reduce the recruitment of osteoclasts by downregulating the release of RANKL. Our results suggest that factors that induce mechanical stress in aging laying hens may not be the same as the associated factors used in aging mammals [33]. Thus, to further understand the bone turnover pathway in hens, studies should include broader techniques to analyze bone by either DNA microarray or RNA sequencing to determine other known or undiscovered transcripts that may have a more prominent role. It is important to note that a gene expression pattern (*i.e*., production of mRNA) is not always directly indicative of protein expression, which actually preforms the cellular function of regulating the transcription of other genes. As a result, we cannot confidently ascribe a function to the genes analyzed simply based on when and where the gene is transcribed [34]. Thus, in an alternative approach, the translated proteins may need to be analyzed to elucidate the effect of mechanical loading on bone. Finally, our method of analyzing whole bone may likely provide some inferences between the specific cell types of bone. Isolating and analysis of each bone-specific cell may provide insight into the specific function of the bone. By measuring each cell separately, we likely are able to include additional controls specific to each cell. In addition, our results indicate that bone remodeling aided by mechanical loading in chickens is probably expressed differently than the corresponding mammalian model. Thus, future studies using chickens as models for the therapeutic treatment of humans for osteoporosis or bone remodeling should be viewed with caution.

## 4. Materials and Methods

### 4.1. Animals

White leghorn females of the Hy-Line W36 strain were raised at Purdue University Poultry Research Farm using standard management and vaccination practices and under the guidelines approved by the Purdue University Animal Care and Use Committee (PACUC Number 10-082; 21 July 2010) [32,35]. Infrared beak trimming was performed at the hatchery [32,35]. Typical starter (0 to 3.9 week of age, CP = 20%, calcium = 1.00% and non-phytate *p* = 0.45%) and grower (4 to 12 week of age, CP = 18.6%, calcium = 1.00% and non-phytate *p* = 0.40%) feed were used. The diet changed at 18 weeks of age, and chickens were switched to a laying hen diet (CP = 18.3%, Ca = 4.20% and non-phytate *p* = 0.30%). They remained on this diet to the end of the study. Diets were fed using the recommendation for nutrients by Hy-Line International (2009–2011) or the National Research Council (1994) [32,35]. Feed and water were provided *ad libitum* for all birds. Feed was also provided on paper lining the cage floor for the first few days following hatching. Two drip nipple drinkers/cage provided a source of water [32,35]. A step-down lighting regimen was used during the pullet phase, where light hours were gradually decreased from 22L:2D at 1 day of age to 12L:12D by 9 weeks of age [32,35]. Beginning at 18 weeks of age, light hours were gradually stepped-up, achieving a photoperiod of 16L:8D by 30 weeks of age, where it remained until termination of the study. Light intensities were set at 32, 2 and 11 lux at 1 day, 1 week and 18 weeks of age, respectively [32,35].

Our study contained four treatment groups defined by access to perches during the pullet and the laying phases. Treatment 1 (control) consisted of chickens with no access to a perch (0 to 71 weeks). Treatment 2 chickens had access to perches only during the egg-laying phase of the life cycle (17 to 71 weeks of age). Treatment 3 chickens had access to perches only during the pullet phase (0 to 16.9 weeks of age). Treatment 4 chickens always had access to perches (0 to 71 weeks of age). For the arrangement of metal round perches within the cage, see [31]. There was enough perching space available at all ages for all chickens to perch at the same time.

Thirty-six chickens from 71 weeks of age were used in this study (*n* = 9 from each treatment). The chickens were euthanized using sodium pentobarbital followed by cervical dislocation [32,35]. Once euthanized, the tibia, femur and phalange were immediately collected, denuded of all muscular and cartilage tissues, rinsed in RNase Away (Ambion, Austin, TX, USA), placed directly on dry ice to freeze and stored in −80 °C until RNA isolation.

### 4.2. Total RNA Isolation and cDNA Synthesis

In the presence of liquid nitrogen and/or dry ice (to keep the bones frozen), the bones were removed from the −80 °C conditions and manually crushed with pliers to form bone fragments. The bone fragments were further processed with a pestle in a stainless steel bowl to produce a bone fragment powder. For total RNA isolation, the bone powder was added to Trisure (Bioline USA Inc., Taunton, MA, USA) following the manufacturer’s guidelines. The bone:Trisure mixture was homogenized using a PRO250 homogenizer, until all tissue was in solution, and subsequently, total RNA was precipitated with isopropanol. Following isopropanol precipitation and ethanol washing, the total RNA pellet was isolated and resuspended with 150–300 μL DEPC water, depending on size of pellet. To assess RNA quality and amount, the final concentration and purity of each RNA pellet were determined by nanospectroscopy. Following RNA isolation, DNase treatment was performed using RQ1 RNase-Free DNase (Promega, Madison, WI, USA) using the manufacturer’s guidelines. DNase-treated RNA was then converted to cDNA using reverse transcriptase and random primers (High Capacity cDNA Reverse Transcription Kit, Applied Biosystems, Foster City, CA, USA).

### 4.3. Real-Time Quantitative PCR and Cloning cDNA for Sequence Validation

Our previous quantitative RT-PCR methods were modified for this study as follows [36]. Real-time quantitative PCR (qPCR) used a series of primer pairs generated for each gene using the NCBI Primer-BLAST (http://www.ncbi.nlm.nih.gov/tools/primer-blast/). All amplicons generated by qPCR were electrophoresed, excised, ligated into pGEMT-easy (Promega), transformed into TOP 10 *E. coli* cells (Invitrogen, Carslbad, CA, USA), screened and sequenced with T7 and Sp6 primers, as previously described [22,36]. The subcloning and DNA sequencing was performed to validate the amplicons generated (Table 1). Using this information, final validated primer pairs were subsequently selected for each locus (Table 1).

Following the manufacturer’s instructions, the qPCR used the Thermo-Fast 96 Detection plate and the DyNamo HS SYBR Green qPCR kit (Thermo Scientific, Waltham, MA, USA) on an Applied Biosystems 7300 Real-Time PCR machine. Each well contained 10 μL of 2× SYBRGREEN Dye Master Mix (Thermo Scientific), 5 μL of 40 ng of cDNA template (10 ng final concentration). Each reaction was performed in triplicate. Five microliters of oligonucleotide primer pair master mix (forward and reverse primer, 500 nM) for each transcript were added to the specific well. The total volume of each well was 20 μL. The qPCR was performed at 95 °C for 2 min followed by 95 °C for 10 s, 62 and 72 °C each at 30 s for 40 cycles. Following the Stage 3 step, the amplification was recorded. Each transcript was examined on separate plates allowing for the maximum amount of samples to be tested with all treatment groups measured.

Glyceraldehyde 3-phosphate dehydrogenase (GAPDH, used as a control) is expressed throughout all tissue used to normalize *C*_t_ values. The seven different genes of interest (GOI) were subtracted from GAPDH using Equation 1 [37]. Analysis of the cycle threshold (*C*_t_) values involved transforming the data using GAPDH (2-change in *C*_t_) [37]. In short, the qPCR was performed on the eight transcripts on each bone in triplicate. The average of the *C*_t_ values for each transcript was determined. Determining the relative quantity without efficiency correction was performed using Equation (2) [38].

Δ*C*_t_ = (GOI *C*_t_) − (GAPDH *C*_t_) (1)

RQ = 2 − (GOI *C*_t_) − (GAPDH *C*_t_) (Relative Quantity) (2)

### 4.4. Statistical Analysis

Statistical Analysis System (SAS) was used to analyze the relative quantity of each gene transcript. The relative quantity of each gene transcript was analyzed by mixed model repeated measures ANOVA (PROC MIXED, SAS Institute Inc., Cary, NC, USA, 2009) with four perch treatments, the repeated measurement factor bone (phalange, tibia and femur) and the treatment × bone interaction as fixed effects, with bird as a random effect [39]. Significant effects were further analyzed by determining differences among least squares means using a Tukey–Kramer adjustment for multiple tests [39].

## 5. Conclusions

This study provided insight into the mechanism of chicken bone turnover in response to perch access (Figure 1). We were able to identify that similar to mammals, SOX9 was not significantly expressed in the bones of aging 71-week-old laying hens. Unlike mammals, where they experience mechanical strain in response to exercise, in hens with a perch, there is no reduction in the RANKL/OPG ratio. Further research into chicken bone turnover should focus directly on bone-specific transcripts, such as alkane phosphatase, whose expression directly correlates to the activity of osteoblasts, as well as calcium sensing receptors that are directly upregulated and downregulated in response to osteoclast activity.

## Appendix

**Table A1:** Mean expression among treatment and bone combinations (least squares mean ± SE). Negative least squares means arise when the measured expression is highly variable among replicates and includes missing values for some replicates (particularly for OPG and RANKL).

Treatment Bone	Control			Perch during Laying			Perch during Pullet			Perch during Pullet and Laying		
	F	P	T	F	P	T	F	P	T	F	P	T
**SOX9**	0.45 ± 0.99	4.29 ± 3.13	0.90 ± 0.74	1.65 ± 0.99	5.96 ± 3.13	0.59 ± 0.74	0.77 ± 0.99	1.41 ± 3.13	1.68 ± 0.74	1.75 ± 0.99	0.58 ± 3.13	1.71 ± 0.74
**PTHLH**	0.75 ± 0.69	1.30 ± 0.58	0.38 ± 0.84	1.77 ± 0.69	0.46 ± 0.58	0.64 ± 0.84	0.25 ± 0.69	0.55 ± 0.58	0.53 ± 0.84	0.10 ± 0.69	0.25 ± 0.58	1.67 ± 0.84
**PTH1R**	0.43 ± 0.15	1.52 ± 0.64	0.30 ± 0.75	0.38 ± 0.15	0.80 ± 0.64	1.25 ± 0.75	0.18 ± 0.15	0.24 ± 0.64	1.89 ± 0.75	0.14 ± 0.15	0.16 ± 0.64	0.40 ± 0.75
**PTH3R**	1.76 ± 0.68	2.99 ± 1.20	0.91 ± 0.67	1.85 ± 0.68	2.23 ± 0.64	1.41 ± 0.67	0.68 ± 0.68	1.28 ± 1.20	1.41 ± 0.67	0.64 ± 0.68	0.56 ± 1.20	2.31 ± 0.67
**OPG**	0.76 ± 1.09	0.18 ± 0.33	0.39 ± 0.39	0.24 ± 1.16	0.70 ± 0.39	0.19 ± 0.41	1.82 ± 1.03	0.76 ± 0.33	0.94 ± 0.37	1.42 ± 1.03	0.08 ± 0.32	0.68 ± 0.36
**RANKL**	1.09 ± 3.85	0.07 ± 0.09	0.23 ± 0.24	7.75 ± 3.84	0.34 ± 0.11	0.26 ± 0.30	−0.66 ± 4.50	0.25 ± 0.09	0.51 ± 0.25	0.45 ± 3.60	0.30 ± 0.09	0.53 ± 0.24
**RANKL/OPG RATIO**	0.23 ± 4.24	−0.04 ± 0.42	0.02 ± 0.53	9.73 ± 4.15	−0.62 ± 0.61	0.05 ± 0.73	−2.67 ± 4.77	−0.62 ± 0.40	0.36 ± 0.57	−1.49 ± 3.79	−0.19 ± 0.41	0.41 ± 0.51
**COL2A1**	0.92 ± 0.19	0.59 ± 0.11	0.68 ± 0.13	1.02 ± 0.19	0.45 ± 0.11	0.69 ± 0.13	0.53 ± 0.19	0.39 ± 0.11	0.51 ± 0.13	0.68 ± 0.19	0.74 ± 0.11	0.84 ± 0.13
